# Xanthine oxidoreductase activity in MASLD: links to lipid metabolism, oxidative stress, and inflammation

**DOI:** 10.3389/fendo.2026.1731772

**Published:** 2026-02-02

**Authors:** Yangyang Cen, Zhiyu Pu, Xuanxuan Zi, Shenglin Peng, Ruihan Liu, Bowen Yang, Yanna Fan, Jianjun Yang, Yi Zhao, Yannan Zhang

**Affiliations:** 1 Department of Nutrition and Food Hygiene, School of Public Health, Ningxia Medical University, Yinchuan, China; 2 Ningxia Key Laboratory of Environmental Factors and Chronic Disease Control, Yinchuan, China; 3 Guangyuan First People’s Hospital, Guangyuan, China

**Keywords:** inflammation, lipid metabolism, MASLD, oxidative stress, XOR

## Abstract

**Aims:**

To evaluate the differences in xanthine oxidoreductase (XOR) activity, metabolomic profiles, markers of oxidative stress, and inflammatory factors between patients with metabolic dysfunction-associated steatotic liver disease (MASLD) and healthy controls, as well as the correlations among these factors.

**Methods:**

A case-control study was conducted involving 54 MASLD patients alongside 54 healthy controls who were matched for age, gender, and ethnicity. Participants underwent comprehensive blood biochemical testing, including liver function, kidney function, glucose, lipid, XOR activity, markers of oxidative stress, and inflammatory factors. Non-targeted metabolomics detection was conducted to identify alterations in the metabolites of MASLD patients.

**Results:**

MASLD patients showed significantly elevated levels of XOR activity, and this increase was positively correlated with significantly altered markers of oxidative stress and inflammatory factors, including increased malondialdehyde levels, tumor necrosis factor-α and interleukin-6. Metabolomic analysis revealed a unique pattern of specific metabolites, including animo acids, sphingolipids, phospholipids, and fatty acids, which were significantly altered in MASLD. A total of 100 metabolites were identified as differentially expressed between MASLD and control groups, with 44 metabolites specifically associated with XOR activity. These metabolites were significantly correlated with lipid profiles, oxidative stress indices, and inflammatory factors.

**Conclusion:**

This study demonstrates significant alterations in XOR activity, lipid metabolism, oxidative stress, and inflammatory reactions in MASLD, as well as the significant association between these factors.

## 1 Introduction

Metabolic dysfunction-associated steatotic liver disease (MASLD), formerly named non-alcoholic fatty liver disease (NAFLD) or metabolic associated fatty liver disease (MAFLD), characterized as a chronic liver condition closely linked to metabolic syndrome, marked by hepatic steatosis and various metabolic abnormalities (1, 2). It can further lead to liver fibrosis and ultimately evolve into metabolic dysfunction-associated steatohepatitis (MASH) and liver cirrhosis, and linked to an elevated risk of disease when influenced by underlying conditions like obesity and type 2 diabetes (3). Over recent years, MASLD has become a significant global public health challenge with rising prevalence worldwide (4). It is currently estimated to affect 39.22% of the global population, with a major impact on clinical and economic burden to society (5, 6). Extensive research has been conducted to investigate the pathogenesis of MASLD and to explore interventions for modifying the disease status, such as adopting of a healthy lifestyle (7, 8). Nonetheless, the exact pathogenic mechanisms of MASLD remain incompletely understood, and there is currently no effective pharmacological treatment that can reverse or cure MASLD.

The pathogenesis of MASLD involves multiple mechanisms, including insulin resistance, mitochondrial dysfunction, oxidative stress, inflammatory response, and metabolic dysregulation (9). Oxidative stress and inflammation play a central role in the progression of MASLD (10). Oxidative stress leads to liver injury by producing reactive oxygen species (ROS), which can harm lipids, proteins, and DNA, leading to inflammation, fibrosis, and ultimately liver cell death (10). Inflammation leads to liver injury by activating immune cells and releasing pro-inflammatory cytokines (11). Chronic inflammation leads to the recruitment of macrophages and neutrophils, which exacerbate tissue damage and promote fibrosis (9). Metabolic dysregulation, including insulin resistance and disturbances in lipid and glucose metabolism, also plays a crucial role in the development and progression of MASLD, contributing to the overall pathophysiology of the disease (12).

Xanthine oxidoreductase (XOR), a member of the metalloflavoenzyme family, is ubiquitously found across species, ranging from prokaryotes to humans, originating from the XOR gene system of an ancestral lineage (13). In mammals, this enzyme can switch between two forms: xanthine oxidase (XO) and xanthine dehydrogenase (XDH). XOR can oxidize a wide range of endogenous metabolites, such as purines, pyrimidines, aldehydes, azapurines, pteridines, and heterocyclic compounds (14, 15). XOR catalyzes the reduction of oxygen to generate ROS, particularly superoxide anions and hydrogen peroxide (16). Consequently, XOR is capable of producing oxidant species that disrupt redox homeostasis and is associated with a variety of biological processes, such as inflammation, cell proliferation, and tumorigenesis (17). The blood XOR activity has been found to be markedly higher both in patients with NAFLD and in animal model of NAFLD (18, 19). Furthermore, it has shown that plasma XOR activity is associated with the risk of hepatic steatosis, independent of insulin resistance and serum UA levels (20). However, the specific relationship between XOR activity and the metabolome of MASLD, as well as the influence of oxidative stress and inflammatory response in it, remains to be fully elucidated.

Given the aforementioned context, this study aims to evaluate the differences in XOR activity, markers of oxidative stress, inflammatory factors, and metabolomic profiles between patients with MASLD and healthy controls, as well as the correlations among these factors. By integrating these factors, this study seeks to provide new insights into the pathophysiology of MASLD, which may contribute to the development of novel strategies for early diagnosis, prevention, and treatment of the disease.

## 2 Materials and methods

### 2.1 Study subjects

This study adopted a case-control design, enrolling MASLD patients from the physical examination center of Kang Yuan Ze Run Hospital between 2021 and 2022. Healthy controls were selected from individuals who underwent routine health check-ups at the same hospital during the same period and were matched to MASLD patients for age, sex, and ethnicity. Written informed consent was obtained from each subject before enrollment. The study was conducted in accordance with the Declaration of Helsinki, and the protocol was approved by the Ethics Committee of Ningxia Medical University (2021-G065) on March 13, 2021.

### 2.2 Diagnostic criteria

The diagnosis of MASLD in this study was based on the diagnostic criteria originally proposed for MAFLD in the 2020 international expert consensus statement (1). In individuals with imaging evidence confirming fatty liver, a diagnosis of MAFLD can be made if any one of the following three conditions is present: I. Overweight or obese: body mass index (BMI) ≥ 23kg/m^2^; II. Type 2 diabetes mellitus; or III. Metabolic dysfunction, defined as meeting at least two of the following criteria: waist circumference (WC) ≥90/80cm in men and women; Blood pressure ≥130/85mmHg; Plasma triglyceride (TG) ≥1.70mmol/L; Plasma high density lipoprotein cholesterol (HDL-C) < 1mmol/L for men and < 1.3mmol/L for women; Pre-diabetes; Homeostasis model assessment of insulin resistance score ≥ 2.5; Plasma high-sensitivity C-reactive protein level > 2 mg/L).

### 2.3 Inclusion and exclusion criteria

MASLD participants were selected considering the following inclusion criteria: Aged 18–65 who meeting the MASLD diagnostic criteria; Willingness to complete questionnaires and undergo necessary examinations and tests. The following exclusion criteria were applied in MASLD participants: Pregnant or breastfeeding women; Known viral hepatitis or other chronic liver diseases; Presence of other serious illnesses, including genetic diseases, cancers, tumors, and serious infectious diseases; Patients who taking XOR inhibitors such as febuxostat and allopurinol; Patients who taking lipid-lowering drugs.

Regarding control participants, the inclusion criteria were: Age, gender and ethnicity matched healthy people during the same period; Willingness to complete questionnaires and undergo necessary examinations and tests. The exclusion criteria are as follows: Known viral hepatitis or other chronic liver diseases; Presence of other serious illnesses, including genetic diseases, cancers, tumors, and serious infectious diseases; Current use of XOR inhibitors such as febuxostat and allopurinol; Current use of lipid-lowering drugs; Pregnancy or breastfeeding; or the presence of any metabolic abnormality, including obesity (BMI ≥ 28.0 kg/m²), dyslipidemia (total cholesterol (T-CHO) ≥ 6.2 mmol/L, TG ≥ 1.7 mmol/L, low density lipoprotein-cholesterol (LDL-C) ≥ 3.4 mmol/L, or HDL-C < 1.0 mmol/L), hyperuricemia (serum uric acid (UA) > 420 μmol/L in men or > 360 μmol/L in women), impaired fasting glucose (fasting blood glucose (FBG) ≥ 6.1 mmol/L), or elevated liver enzymes (alanine aminotransferase (ALT) > 35 U/L or aspartate aminotransferase (AST) > 40 U/L).

### 2.4 Data and sample collection

Participants completed a structured questionnaire that included demographic information (age, sex and nationality), living habits (smoking and alcohol consumption), and medical history. Weight and height were measured to calculate BMI. WC, systolic blood pressure (SBP) and diastolic blood pressure (DBP) were also evaluated. Abdominal ultrasound scans were performed by experienced doctors to assess hepatic steatosis and other structural abnormalities. Venous blood was taken from participants after overnight fasting and collected into two types of tubes: one type containing an anticoagulant for plasma collection and another type without any additives for serum collection. The blood in the serum collection tubes was allowed to clot for 30 minutes at room temperature before centrifugation. Both sets of samples were then centrifuged at 3000 rpm for 10 minutes. The resulting supernatants (plasma and serum) were carefully removed and stored at ultra-low temperature refrigerators until analysis.

### 2.5 Biochemical testing

Serum levels of ALT, AST, blood urea nitrogen (BUN), creatinine, TG, T-CHO, HDL-C, LDL-C, UA, FBG were measured by an automatic biochemical analyzer (Shanghai Aino Electronics Co., LTD, Healforce MOL300, Shanghai, China).

The oxidative activity of XOR in serum samples was measured using a colorimetric method with commercial kits (Nanjing Jiancheng, Nanjing, China).

Serum oxidative stress index including glutathione peroxidase (GSH-Px), malondialdehyde (MDA) and superoxide dismutase (SOD) were determined by commercial test kits (Nanjing Jiancheng, Nanjing, China).

Plasma proinflammatory factor concentrations, including interleukin (IL)-1β, IL-6, and tumor necrosis factor (TNF)-α were measured using ELISA kits (Jianglaibio Co., LTD, Shanghai, China).

### 2.6 Serum metabolomics study

Metabolites extraction: 100uL of serum sample was transferred to EP tube. Then, prepare 400uL solution mixed with equal volumes of methanol and acetonitrile, and add it to the EP tube. After thoroughly mixing and centrifuging, the supernatant to be analysis was obtained.

Metabolites detection: The metabolomics analysis was carried out in a Waters ACQUITY UPLC BEH Amide system (Thermo Fisher Scientific) equipped with the Xevo G2-S Q-TOF MS system (Thermo Fisher Scientific).

The initial data were transformed into the mzXML format utilizing ProteoWizard, and subsequently analyzed with a proprietary R script that leverages XCMS for the identification, extraction, alignment, and aggregation of peaks. For metabolite identification, a custom MS2 database known as BiotreeDB was employed, applying a threshold of 0.3.

### 2.7 Statistical analysis

Normally distributed continuous data were reported as the mean with standard deviation, while non-normally distributed continuous data were depicted using the median and interquartile range. Categorical variables were presented as counts and percentages. For the analysis of continuous data, Student’s t-tests or Mann–Whitney U tests were conducted, and for categorical data, chi-square tests were applied. Spearman’s correlation analysis was used to calculate possible relationships among demographic data, blood biochemical parameters and metabolites. For all analyses, *p*-value < 0.05 was considered statistically significant. The correlation analysis and visualization were performed using R, the remaining statistical analyses were performed using IBM SPSS 21 software (SPSS Inc., Chicago, IL, USA).

Multivariate analysis of the metabolomics dataset was performed using SIMCA-P+ version 14.0 (Umetrics, Umea, Sweden). The data were preprocessed by scaling and logarithmic transformation to reduce the influence of noise and high variance in the variables. To discern group differences and pinpoint metabolites with significant alterations, we employed supervised orthogonal partial least squares-discriminant analysis (OPLS-DA). This approach was validated using a 7-fold cross-validation to ascertain R² and Q² metrics, indicating goodness of fit and predictability, respectively. Robustness and predictive ability were further assessed through 200 permutation tests, with lower Q² intercept values suggesting less overfitting. Additionally, the significance of the first principal component’s variable importance in the projection (VIP) was determined in the OPLS-DA model. Metabolites were deemed to have undergone significant changes if they had a VIP score above 1 and a *p*-value less than 0.05, as assessed by Student’s t-test. Metabolite concentration differences between the groups were also evaluated in terms of log fold change. In addition, MetaboAnalyst 5.0 platform (http://www.metaboanalyst.ca/) was used for pathway enrichment analysis. Statistically significant were considered as *p* < 0.05.

## 3 Results

### 3.1 Characteristics of study participants

Data was obtained from 108 participants, including 54 individuals diagnosed with MASLD and 54 healthy controls. The average age of participants in the MASLD group was 37.39 ± 8.07 years, which was comparable to the control group’s average age of 37.35 ± 7.94 years (*p* = 0.98). The majority of participants were of Han nationality, comprising 79.63% of the control group and 85.19% of the MASLD group (*p* = 0.62). The study included an equal number of male and female participants in both the control and MASLD groups, ensuring a balanced gender distribution. BMI, WC, SBP and DBP were significantly elevated in the MASLD group compared to controls (*p*<0.05). Detailed demographic and clinical data were shown in **Table 1**.

**Table 1:** Demographic characteristics of individuals diagnosed with MASLD and healthy subjects.

Parameter	Control (n=54)	MASLD (n=54)	*p* Value
Age (years)	37.35 ± 7.94	37.39 ± 8.07	0.98
Male (n, %)	30 (55.56%)	30 (55.56%)	1
Han Nationality (n, %)	43 (79.63%)	44 (85.19%)	0.62
Anthropometric assessments			
BMI (kg/m^2^)	22.10 ± 2.47	26.44 ± 3.39	<0.01
Waist (cm)	78.47 ± 5.27	91.83 ± 9.23	<0.01
Blood pressure			
SBP (mm Hg)	115 (108, 122)	126 (120, 130)	<0.01
DBP (mm Hg)	77 (72, 83)	82 (75, 90)	0.02
Living habits			
Smoking (n, %)	17 (31.48%)	21 (38.89%)	0.42
Alcohol Drinker (n, %)	24 (44.44%)	30 (55.56%)	0.24

Values are presented as mean ± SD, median (interquartile range), or count (%). BMI, body mass index; DBP, diastolic blood pressure; SBP, Systolic blood pressure; WC, waist circumference.

### 3.2 Blood biochemical parameters

The blood biochemical parameters of the individuals diagnosed with MASLD were contrasted against those from the healthy control subjects (**Table 2**), revealing significantly higher levels of ALT and AST in the MASLD group (p < 0.01). Similarly, TG, T-CHO and LDL-C were also significantly elevated in the MASLD group (*p* < 0.01), while HDL-C was significantly lower (*p* < 0.01). FBG tended to be higher in the MASLD group (5.61 ± 1.82 mmol/L) compared to controls (5.26 ± 0.66 mmol/L), but this difference was not statistically significant (*p* > 0.05).

**Table 2:** Blood biochemical parameters of individuals diagnosed with MASLD and healthy subjects.

Parameter	Control (n=54)	MASLD (n=54)	*p* Value
Liver enzyme levels			
ALT (U/L)	18.70 (13.00, 23.80)	33.00 (20.90, 50.13)	<0.01
AST (U/L)	20.10 (17.50, 22.20)	22.85 (19.28, 29.18)	<0.01
Lipid levels			
TG (mmol/L)	1.09 (0.84, 1.54)	2.47 (1.67, 3.37)	<0.01
T-CHO (mmol/L)	4.50 ± 0.66	5.16 ± 1.04	<0.01
HDL-C (mmol/L)	1.48 ± 0.31	1.35 ± 0.28	<0.01
LDL-C (mmol/L)	2.55 ± 0.44	2.87 ± 0.58	<0.01
FBG (mmol/L)	5.26 ± 0.66	5.61 ± 1.82	0.50
Renal function markers			
BUN (mmol/L)	4.60 (3.80, 5.40)	4.25 (3.80, 5.65)	0.60
Creatinine (μmol/L)	60.57 ± 10.20	59.13 ± 1.81	0.50
XOR and product			
XOR (U/L)	4.35 ± 0.79	5.28 ± 1.70	<0.01
UA (μmol/L)	313.49 ± 72.60	399.48 ± 88.64	<0.01
Oxidative stress indicators			
GSH-Px (mU/mL)	246.16 (211.53, 263.75)	227.78 (211.13, 246.16)	0.04
MDA (nmol/mL)	2.34 (2.00, 3.31)	3.20 (2.49, 4.22)	0.01
SOD (U/mL)	12.10 (11.81, 12.49)	11.81 (11.50, 12.22)	0.02
Inflammatory factors			
TNF-α (pg/mL)	21.17 (10.96, 29.99)	62.29 (28.91, 148.15)	<0.01
IL-1β (pg/mL)	44.36 (17.12, 103.51)	112.17 (60.95, 136.66)	<0.01
IL-6 (pg/mL)	7.96 (5.00, 16.54)	13.98 (6.79, 25.94)	<0.01

Values are presented as mean ± SD or median (interquartile range). ALT, Alanine aminotransferase; AST, aspartate aminotransferase; BUN, blood urea nitrogen; FBG, fasting blood glucose. HDL-C, high density lipoprotein-cholesterol; IL, interleukin. LDL-C, low density lipoprotein-cholesterol; TG, triglyceride; TNF, tumor necrosis factor. T-CHO, total cholesterol; UA, uric acid; XOR, Xanthine oxidase.

XOR activity and UA concentrations were markedly elevated in individuals with MASLD as opposed to the control group (*p* < 0.01). Regarding oxidative stress markers, levels of GSH-Px and SOD were considerably reduced in the MASLD patients compared to controls (*p* < 0.05), whereas MDA levels were notably increased (*p* < 0.01). Furthermore, concentrations of all proinflammatory cytokines (TNF-α, IL-1β, and IL-6) were significantly elevated in the MASLD group versus the control group (*p* < 0.01).

### 3.3 Associations between XOR activity and other metabolic health parameters

**Figure 1:**
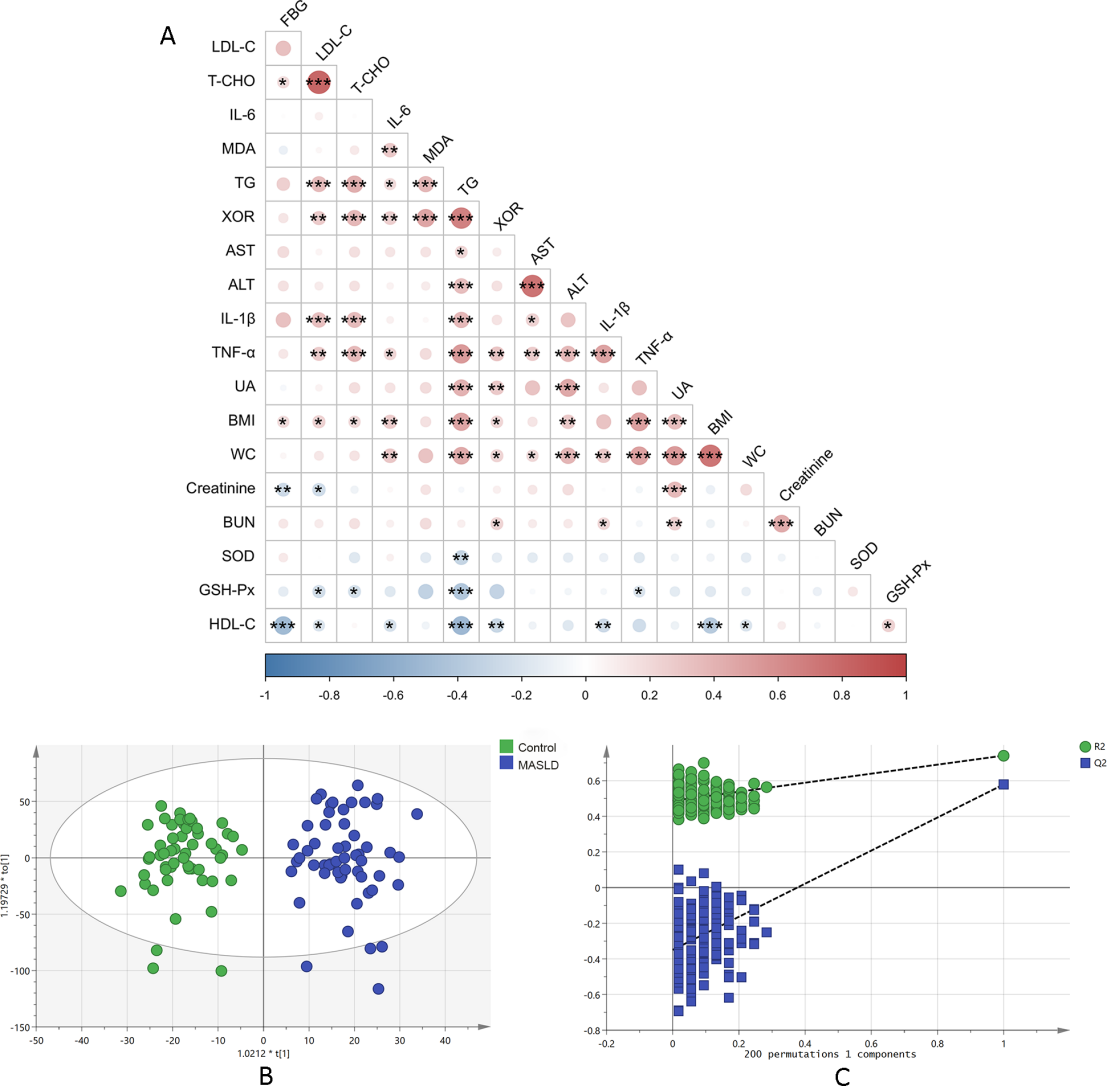
Correlation of XOR and metabolic health parameters **(A)**, OPLS-DA scores plot **(B)** and permutation tests **(C)** based on UHPLC-MS data of serum samples obtained from control and MASLD patients. Correlations were calculated using Spearman’s correlation coefficient. ALT, Alanine aminotransferase; AST, aspartate aminotransferase; BMI, body mass index; BUN, blood urea nitrogen; FBG, fasting blood glucose. HDL-C, high density lipoprotein-cholesterol; IL, interleukin. LDL-C, low density lipoprotein-cholesterol; TG, triglyceride; TNF, tumor necrosis factor. T-CHO, total cholesterol; UA, uric acid; WC, waist circumference; XOR, Xanthine oxidase. *p < 0.05, **p < 0.01, ***p < 0.001.

To identify correlations between XOR and other metabolic health parameters such as BMI, WC, blood biochemical index, oxidative stress index, and inflammatory index, we performed Spearman’s correlation analysis (**Figure 1A**). BMI, WC, TG, T-CHO, LDL-C, BUN, UA, MDA, TNF-α and IL- 6 displayed positive correlations with XOR activity (*p* < 0.05), while HDL-C showed a negative correlation with XOR activity (*p* < 0.05). In addition, TG, T-CHO, BMI and WC also showed significant correlations with other metabolic health parameters.

### 3.4 Metabolomic alterations in MASLD

Using untargeted metabolomics, the serum metabolites were examined. A total of 13149 features were obtained from positive and negative scan models of UHPLC-MS/MS analysis.

We performed OPLS-DA model to reveal the substantial differentiation in the metabolomic profiles between the two groups. The OPLS-DA score plot showed a distinct separation between the two groups, indicating that the model successfully discriminated between them (**Figure 1B**, R^2^X=0.297, R^2^Y=0.895, Q^2^ = 0.654). Permutation testing with 200 iterations confirmed the validity of the model, as the original model’s Q² value was significantly higher than those obtained from the permuted models (**Figure 1C**).

**Figure 2:**
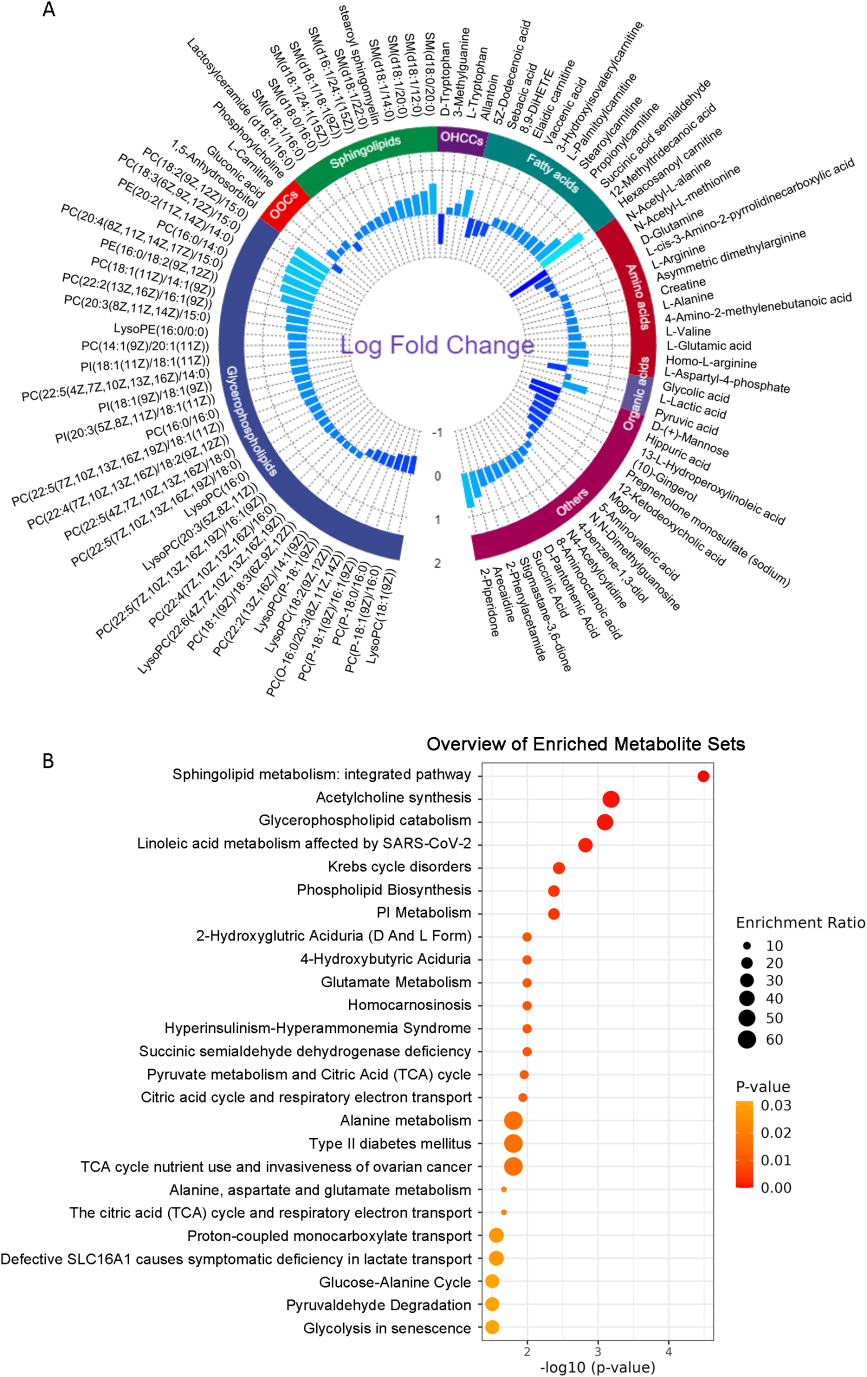
Significant metabolites and enrichment analysis. **(A)** The circular histogram of 100 significant metabolites (VIP > 1, *p* < 0.05). The inner-circle shows the log fold change values of these metabolites. **(B)** Results of enrichment analysis of significant metabolites. LysoPC, lysophosphatidylcholine; PC, phosphatidylcholine; PE, phosphatidylethanolamine; PI, phosphatidylinositol; OHCCs, organoheterocyclic compounds; OOCs, organic oxygen compounds; SM, sphingomyelin.

A total of 100 distinct metabolites were recognized, exhibiting differences between the MASLD cohort and the control group. (**Figure 2A**). The altered metabolites are categorized into several main classes including glycerophospholipids [e.g. lysophosphatidylcholine (LysoPC) (18:1(9Z)] and phosphatidylcholine (PC) (18:2(9Z,12Z)/15:0)), sphingolipids [e.g. sphingomyelin (SM) (d18:1/16:0 and SM (d18:0/20:0)], organic oxygen compounds (e.g. 1,5-Anhydrosorbitol), fatty acids (e.g. 5Z-Dodecenoic acid and Hexacosanoyl carnitine), amino acids (e.g. N-Acetyl-L-alanine and L-Aspartyl-4-phosphate), organic acids (e.g. Glycolic acid), and other compounds.

Based on the identified differential metabolites, an enrichment analysis was conducted to facilitate further biological interpretation and reveal the key pathways involved in MASLD. The findings revealed that the distinct metabolites predominantly participated in 39 metabolic pathways (*p* < 0.05), the top 5 enriched metabolic pathways including sphingolipid metabolism, acetylcholine synthesis, glycerophospholipid catabolism, linoleic acid metabolism affected by SARS-CoV-2, and Krebs cycle disorders (**Figure 2B**).

### 3.5 Associations between metabolites, XOR activity, oxidative stress, and inflammation

**Figure 3:**
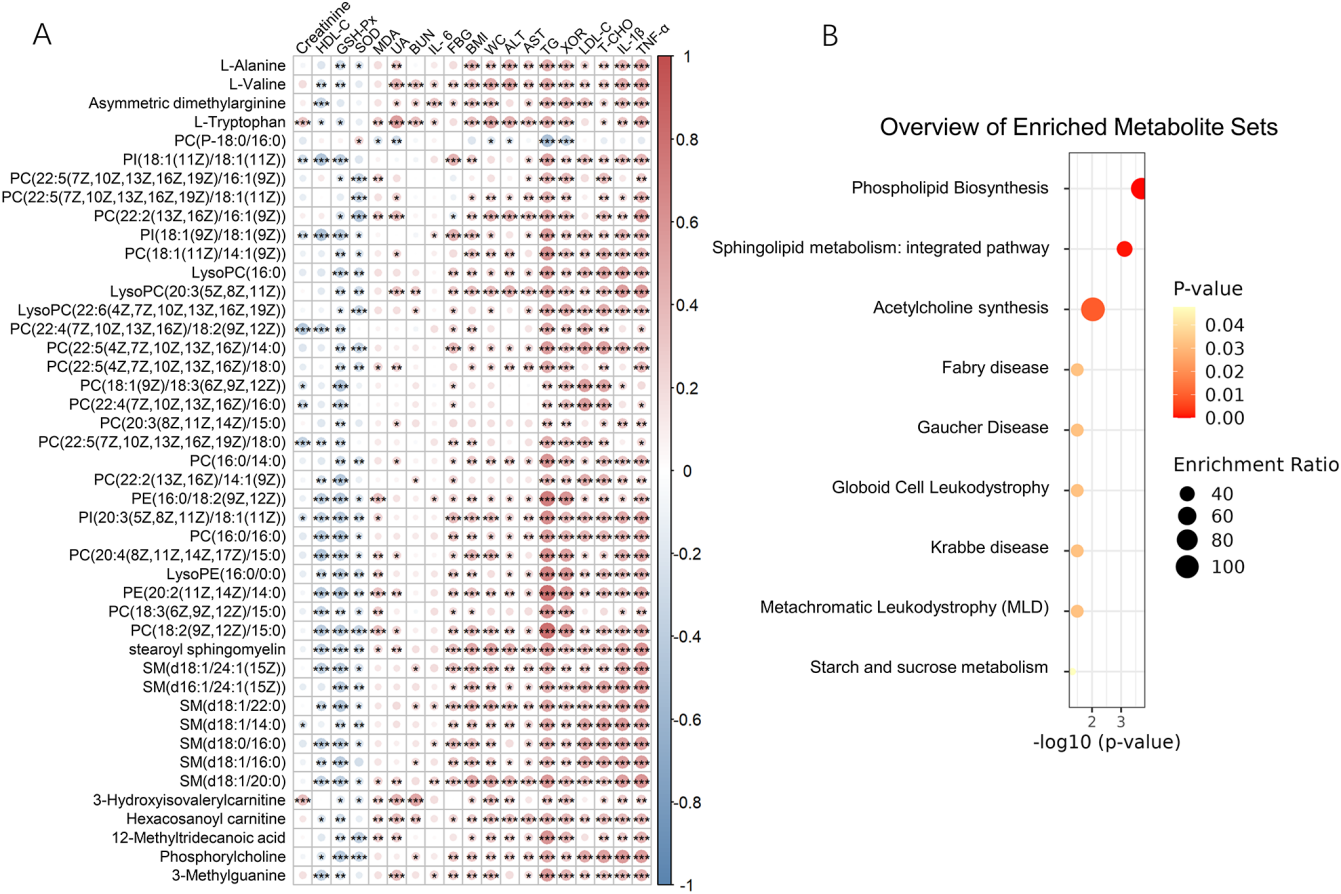
Correlation of XOR-related metabolites and metabolic health indicators **(A)** and metabolites enrichment analysis **(B)**. Correlations were calculated using Spearman’s correlation coefficient. ALT, Alanine aminotransferase; AST, aspartate aminotransferase; BMI, body mass index; BUN, blood urea nitrogen; FBG, fasting blood glucose. HDL-C, high density lipoprotein-cholesterol; IL, interleukin. LDL-C, low density lipoprotein-cholesterol; LysoPC, lysophosphatidylcholine; PC, phosphatidylcholine; PE, phosphatidylethanolamine; PI, phosphatidylinositol; SM, sphingomyelin; TG, triglyceride; TNF, tumor necrosis factor. T-CHO, total cholesterol; UA, uric acid; WC, waist circumference; XOR, Xanthine oxidase. *p < 0.05, **p < 0.01, ***p < 0.001.

To further document whether the change of metabolome in MASLD related to XOR, we performed Spearman correlation analyses using the differential metabolites and XOR activity. A significant correlation (|r| > 0.3, *p* < 0.05) was identified between the changes in differential metabolites and XOR activity. Results revealed that 44 metabolites remained significantly associated with XOR activity. Nearly all the metabolites are lipids, including glycerophospholipids, sphingolipids and fatty acids. Correlation analyses were performed to assess their potential relationships with indices of oxidative stress, inflammatory markers, and other clinical parameters (**Figure 3A**). These metabolites displayed significant correlations with lipid profiles, oxidative stress indices, and inflammatory factors. Specifically, most metabolites showed significant positive correlation with TG, T-CHO and LDL-C, while exhibited a significant negative correlation with HDL-C. Furthermore, the majority of these metabolites exhibited a significant positive correlation with MDA, and a negative correlation with GSH-Px and SOD. In addition, most of these metabolites were significantly positively correlated with pro-inflammatory cytokines such as TNF-α, IL-1β and IL-6.

Enrichment analysis was performed to reveal the most relevant pathways. As shown in **Figure 3B**, the most enriched metabolic pathways were phospholipid biosynthesis, sphingolipid metabolism and acetylcholine synthesis.

## 4 Discussion

Our integrated analysis reveals a significant association between elevated XOR activity, altered lipid metabolism, heightened oxidative stress, and chronic inflammation in patients with MASLD. We further identify a distinct set of XOR-associated metabolites, primarily glycerophospholipids, sphingolipids, and fatty acids, that correlate with markers of hepatic injury, oxidative damage, and pro-inflammatory cytokines. These findings highlight XOR as a potentially important contributor to the metabolic and inflammatory disturbances characteristic of MASLD, offering new insights into its pathophysiology and potential avenues for biomarker and therapeutic development.

### 4.1 XOR’s role in oxidative stress, lipotoxicity, and inflammation driving MASLD progression

The elevated XOR activity in MASLD patients in this study aligns with previous studies demonstrating increased XOR activity in MASLD (18, 20). XOR is an important source of ROS, and its enhanced activity leads to elevated ROS levels under a variety of pathological conditions, including NAFLD (19). We also observed a significant decrease in the activity of antioxidant enzymes such as GSH-PX and SOD. This indicates that the body is undergoing a state of oxidative stress, in which the production of ROS exceeds the clearance capacity of the antioxidant system. The reduction of GSH-PX and SOD may be due to their consumption during ROS clearance, or because their expression or activity is inhibited. Critically, excessive ROS can damage mitochondrial respiratory chain complexes and dissipate the mitochondrial membrane potential, thereby impairing fatty acid β-oxidation (21). This metabolic defect leads to the accumulation of free fatty acids, which are subsequently diverted toward the synthesis of triglycerides and cytotoxic lipid species such as diacylglycerols and ceramides (22). Studies have shown that lipotoxic substances, such as saturated fatty acids and LPC, can activate the Toll-like receptor 4 (TLR4) and c-Jun N-terminal kinase (JNK) signaling pathways, leading to the release of pro-inflammatory cytokines including IL-6, IL-18, and TNF-α (23). Moreover, ROS can further promote inflammation by inducing lipid peroxidation, particularly through oxidation of the double bonds in polyunsaturated fatty acids, as well as causing protein oxidation and DNA damage (24). The severity of NAFLD has been linked to increases in oxidative stress and a proinflammatory state (25). Studies have shown that NAFLD is associated with the massive release and upregulation of pro-inflammatory mediators such as IL-1β, IL-6, and TNF-α (25, 26). Chronic inflammation, driven by the activation of immune cells and the release of pro-inflammatory cytokines, exacerbates tissue damage and promotes fibrosis (11, 27). The Significantly elevated inflammatory factors in our study further underscore the importance of inflammation in the progression of MASLD. In addition, the significant correlation between oxidative stress, XOR and inflammatory factors (shown in **Figure 1A**) also indicates that XOR plays an important role in oxidative stress and inflammation driving MASLD progression. Mechanistically, elevated XOR activity fuels ROS production, which promotes lipid peroxidation and leads to the formation of cytotoxic aldehydes (e.g., MDA). This oxidative insult exacerbates mitochondrial dysfunction in hepatocytes, impairing fatty acid β-oxidation and promoting intracellular lipid accumulation—a hallmark of lipotoxicity. Concurrently, XOR-derived ROS activate key inflammatory pathways, including TLR4 and JNK inflammasome, leading to the upregulation of pro-inflammatory cytokines such as IL-6 and TNF-α, which further perpetuate hepatic injury and fibrosis.

### 4.2 Metabolomic alterations in MASLD

Metabolomics has become a powerful tool for understanding the complex metabolic alterations in diseases (28–30). Many researchers have performed metabolomics studies to investigate metabolic fingerprints, pathway alterations, and biomarkers in MASLD (31–33). Our study employed untargeted metabolomics to identify serum metabolite changes in patients with MASLD compared to healthy controls. A comprehensive metabolomic analysis of UHPLC-MS/MS combined with multivariate statistics revealed a clear separation between MASLD and the control group, confirming a unique metabolic profile associated with MASLD.

Our analysis identified 100 differential metabolites that were significantly altered in MASLD compared to healthy controls. These metabolites include glycerophospholipids, sphingolipids, organic oxygen compounds, fatty acids, amino acids, organic acids, and others. Notably, the altered metabolites were primarily lipids, which aligns with the known lipid (TC, TG and LDL-C) accumulation in MASLD (shown in **Table 2**). The identification of specific metabolites, such as hexacosanoyl carnitine, PC (18:2(9Z,12Z)/15:0) and SM (d18:0/20:0), provides insights into the metabolic disturbances that underlie MASLD pathogenesis. Enrichment analysis of these differential metabolites revealed that they were mainly involved in sphingolipid metabolism, acetylcholine synthesis, glycerophospholipid catabolism, linoleic acid metabolism and other metabolisms. These findings suggest that MASLD is characterized by widespread metabolic dysregulation, particularly affecting lipid metabolism, energy production, and neurotransmitter synthesis. This observation aligns with previous reports of lipid metabolism disorders in NAFLD patients, specifically highlighting disturbances in sphingolipid metabolism and glycerophospholipid catabolism (31, 34, 35). For example, 20 plasma metabolites, primarily sphingolipids and phospholipids, were identified as a metabolic signature that could discriminate NASH and steatosis in the progression of NAFLD (35). These changes could potentially serve as biomarkers for early detection and monitoring of NAFLD progression.

### 4.3 Metabolite-XOR activity correlation

XOR has been recognized not only as a key enzyme in purine catabolism but also as a novel regulator of lipid metabolism. It acts upstream of peroxisome proliferator-activated receptor gamma (PPARγ) during adipogenesis, and its knockdown markedly suppresses both adipocyte differentiation and PPARγ activation *in vitro*. Consistent with this, XOR-deficient mice exhibit approximately a 50% reduction in adipose tissue mass compared with wild-type littermates (36), underscoring XOR’s systemic role in lipid homeostasis. Importantly, our research reveals strong associations between XOR activity and differential metabolites in MASLD patients, suggesting that XOR may also contribute to hepatic lipid metabolism dysregulation. The metabolites significantly correlated with XOR activity (|r| > 0.3, p < 0.05) include amino acids, glycerophospholipids, sphingolipids, fatty acids. Enrichment analysis of metabolites significantly associated with XOR activity revealed a number of pathways that may be involved in the pathophysiology of MASLD, mainly focusing on lipid metabolism (e.g. sphingolipid metabolism, phospholipid biosynthesis, acetylcholine synthesis, **Figure 3B**). The centrality of XOR in these metabolic disturbances may be partly attributed to its capacity to generate ROS, which can influence signaling pathways that regulate lipid synthesis and storage. Further reinforcing XOR’s metabolic relevance, previous studies have shown that plasma XOR activity is independently associated with levels of adipokines in the general population (37). Moreover, when XOR binds to occludin and butyrophilin subfamily 1 member A1 (BTN1a1), it serves as a target of Src kinase and promotes lipid secretion (38). Collectively, these findings highlight the importance of XOR in the metabolic dysregulation characteristic of MASLD, especially in lipid metabolism.

### 4.4 Metabolite-oxidative stress correlation

The significant correlations observed between the metabolites (most are lipids) and oxidative stress indices in this study (**Figure 3A**) suggest that the dysregulation of these metabolites may be influenced by oxidative stress in MASLD. Oxidative stress is known to play a pivotal role in the progression of MASLD, contributing to liver damage and inflammation (10). Additionally, it plays a causative role in adipose tissue deposition, involving preadipocyte proliferation, adipocyte differentiation, and growth (39). Oxidative stress has profound impacts on lipid metabolism, influencing lipid synthesis, breakdown, and storage through various pathways (40). For instance, oxidative stress can activate or inhibit key enzymes involved in lipid metabolism, such as ACC and CPT1, which control the rate of fatty acid synthesis and fatty acid β-oxidation (41, 42). Conversely, disorders in lipid metabolism, such as an excess of free fatty acids, can enhance mitochondrial β-oxidation, leading to increased oxidative stress and promoting the accumulation of triacylglycerols within lipid droplets (43). In addition, lysophosphatidylcholine causes endothelial cell injury by inducing nitric oxide (NO) production via oxidative stress (44). Therefore, the correlation of these metabolites with oxidative stress indices provides further evidence of their potential involvement in the pathogenesis of MASLD.

### 4.5 Metabolite-inflammatory factors correlation

The relationship between the metabolism of lysoPC and inflammation has been reported. For example, it has been reported that lysoPC synthesis pathways were positively correlated with circulating IL-1β and TNF-α (45). In the current study, we found that three types of lysoPC (LysoPC(16:0), LysoPC(20:3(5Z,8Z,11Z)), LysoPC(22:6(4Z,7Z,10Z,13Z,16Z,19Z))) are positively related to IL-1β and TNF-α in inflammatory cytokines (**Figure 3A**). The relationship between lysoPC synthesis and inflammation is currently considered to be complex. LysoPC can induce the production of pro-inflammatory cytokines through various signaling pathways, including platelet-activating factor (PAF) receptor-dependent mechanisms and activation of Interferon-gamma (IFN-γ), p38 Mitogen-Activated Protein Kinase (p38 MAPK), and JNK pathways (46). Besides, excessive lysoPC in plasma can be hydrolyzed, producing choline and pro-inflammatory lysophosphatidic acid (47). However, it can also reduce inflammation by inhibiting the release of pro-inflammatory cytokines and increasing the expression of SOD (48). The dual effects of LysoPC are likely mediated by TLR ligands and the specific structure of the fatty acyl chain (49).

### 4.6 Clinical implications

Furthermore, XOR activity demonstrates potential as a biomarker reflective of the core pathogenic mechanisms in MASLD. Compared to conventional liver injury markers like ALT and AST, XOR activity is directly linked to oxidative stress, lipid dysregulation, and inflammation - integral components of MASLD pathophysiology (50, 51). Its elevation may precede significant hepatocellular damage, offering complementary value for early detection and disease monitoring (52). Therefore, XOR activity could serve as a novel biomarker to aid in the diagnosis and assessment of MASLD, warranting further validation in longitudinal studies. Importantly, our metabolomic data reveal correlations between XOR activity and specific lipotoxic metabolites, including glycerophospholipids, sphingolipids and fatty acids, suggesting that these molecules may serve as noninvasive indicators of the underlying pathophysiological state associated with XOR activity. Thus, XOR activity, either alone or in combination with its associated metabolic signatures, could support both risk stratification and the selection of patients for targeted intervention in MASLD, warranting validation in prospective studies.

### 4.7 Strengths and limitations

This study provides novel insights into the association between XOR activity and metabolites, highlighting the potential role of oxidative stress and inflammatory responses in the pathogenesis of MASLD. The study includes comprehensive biochemical testing and detailed assessments of metabolic parameters, which strengthens the reliability of the findings.

This study has several limitations. First, its cross-sectional design and relatively small sample size preclude causal inference and may limit the generalizability of the findings. Second, we did not comprehensively account for several potential confounders, including dietary intake, undetected preclinical metabolic conditions (e.g., subclinical insulin resistance), and the use of nonspecific plasma oxidative stress markers that may not directly reflect hepatic oxidative stress. Third, future studies are needed to validate and extend our observations, such as randomized controlled trials to assess XOR inhibition in MASLD, larger multi-center cohorts to confirm associations, and studies integrating plasma biomarkers with liver tissue analysis or fibrosis stratification tools (e.g., FIB-4) in histologically confirmed cohorts to evaluate the prognostic relevance of XOR activity.

## 5 Conclusions

This study demonstrates significant alterations in XOR activity, lipid metabolism, oxidative stress, and inflammatory reactions in MASLD, as well as the significant association between these factors. These findings provide valuable insights into the complex interplay between metabolic disturbances and liver disease, suggesting potential targets for the diagnosis, prevention, and treatment of MASLD. Further research is needed to elucidate the specific mechanisms by which these metabolites contribute to the pathogenesis of MASLD and to determine their potential as biomarkers and therapeutic targets.

## Data Availability

The original contributions presented in the study are included in the article/supplementary material. Further inquiries can be directed to the corresponding author/s.
